# Two dimensions of pain acceptance: divergent links to opioid misuse and readiness to change

**DOI:** 10.3389/fpsyg.2026.1879837

**Published:** 2026-10-16

**Authors:** Lara Rose LoBrutto, Emily Donovan, Sophia Burke, Wally Smith, Dace Svikis, Taylor Crouch

**Affiliations:** 1 Department of Psychology Virginia Commonwealth University, Richmond, VA, United States; 2 VA James A Haley Hospital, Tampa, FL, United States; 3 Department of Psychiatry Virginia Commonwealth University School of Medicine, Richmond, VA, United States

**Keywords:** chronic pain, long-term opioid therapy, opioid misuse, pain acceptance, stages of change

## Abstract

More than 13 million Americans remain on long-term opioid therapy for chronic pain (CP) despite well-established risks and no quality evidence of long-term benefits. Pain acceptance, defined as willingness to experience pain and engage in valued life activities despite pain, has been consistently linked to improved pain outcomes and more recently identified as a protective factor against opioid misuse. This study examined the relationship between both factors of pain acceptance and opioid misuse behaviors and readiness to change opioid use among opioid-using individuals with CP. A community sample of adults with a variety of CP conditions using opioid medications (*N* = 157) were recruited through ResearchMatch.org. Participants completed self-report questionnaires, including the Chronic Pain Acceptance Questionnaire (CPAQ), Prescription Opioid Misuse Index (POMI), Brief Pain Inventory (BPI), and single-item Visual Analog Scale (VAS) measures of readiness and confidence to discontinue opioid use. Two CPAQ subscales (activity engagement and pain willingness) and pain severity were entered into a logistic regression predicting POMI score and multiple linear regression models predicting readiness and confidence to reduce opioid use. All three models were significant (*p* < 0.05). Activity engagement was associated with greater odds of opioid misuse, whereas pain willingness was associated with lower odds of misuse. In contrast, higher activity engagement and lower pain willingness predicted higher readiness and confidence to taper opioid use. Findings demonstrate nuances in the dimensions of pain acceptance and support the value of further exploring activity engagement and pain willingness in efforts to reduce opioid-related risks.

## Background

The opioid epidemic continues to be a national public health crisis in the United States, with over 105,000 prescription and non-prescription deaths from overdose in 2023 (National Institute on Drug Abuse, 2024). Opioid prescriptions in medical settings were responsible for the first wave of the crisis (Madras, 2017), with nearly half of treatment-seekers reporting initial prescription by a provider (Cicero et al., 2017). In response to the crisis, the Centers for Disease Control and Prevention issued updated opioid treatment recommendations in 2022 with more detailed guidelines on prescribing, alternative therapies, and recommendations for tapering (Dowell et al., 2022). Still, misuse of prescribed opioids continues to affect millions of Americans (Substance Abuse and Mental Health Services Administration, 2025), with long-term use associated with greater odds of all-cause mortality (Hurtado et al., 2024).

At least one in five American adults experiences chronic pain (CP), an umbrella term used to describe any pain that persists for greater than 3 months (Yong et al., 2022). CP may result from a chronic health condition (e.g., cancer, rheumatoid arthritis) or lack clear etiology and may be site-specific (e.g., low back or neck pain) or widespread (Hadlandsmyth et al., 2024). Social consequences (e.g., absenteeism, social disability), poor mental health outcomes (e.g., anxiety, depression), and reductions in ability to complete tasks of daily living are all associated with CP (De La Rosa et al., 2023; Ueda et al., 2024). CP may be treated via psychological, pharmacological, and alternative modalities and is often best addressed through multiple channels simultaneously (Nijs et al., 2024).

In spite of viable alternatives, long-term opioid therapy (LTOT) remains a common treatment for individuals with CP and is associated with significant consequences. LTOT is most commonly defined as more than 10 prescriptions of 90 or more days or a prescription of more than 120 days in a 6-month period (Karmali et al., 2020). Up to 25% of individuals with chronic non-cancer pain engage in LTOT (Karmali et al., 2020). Risks of LTOT often outweigh the benefits, with consequences including tolerance, cardiac and respiratory risk, hyperalgesia, and depression (Lembke et al., 2016; Manhapra, 2022). LTOT commonly results in physiological dependence, which should be distinguished from opioid misuse and opioid use disorder. More recently, the construct of complex persistent opioid dependence has been proposed to describe patients who develop opioid dependence and difficulty tapering because of opioid-induced hyperalgesia, withdrawal-related symptom amplification, or worsening pain, despite using opioids largely as prescribed (Manhapra, 2022). In addition to the risks of complex persistent opioid dependence, approximately one in four long-term opioid users are at risk of opioid misuse, referring to use of prescription drugs without a prescription or in a way other than prescribed, and about one in 10 are at risk of opioid addiction, or “patterns of continued use with experience of, or demonstrated potential for, harm” (Vowles et al., 2015). Beyond established risks, there is limited evidence of efficacy of LTOT, with close to half of individuals on LTOT reporting dissatisfaction with their pain care (Krebs et al., 2020).

Stages of Change and self-efficacy are both concepts integral to the Transtheoretical Model of Health Behavior Change (TTM), which was established to outline the factors and processes that facilitate behavioral change (Prochaska and Velicer, 1997). Stages of Change describes five stages of “readiness” ranging from pre-contemplation to maintenance that individuals cycle through when attempting to reduce or quit an addictive behavior, and self-efficacy describes one’s “confidence” in their ability to change (Prochaska and Velicer, 1997). These two constructs are used widely in behavior change research and clinical practice and has been applied to numerous health contexts, including in the study of CP management (Ankawi et al., 2019) and substance use (Harrell et al., 2013). In particular, while their use is limited thus far in this context, concepts of readiness and self-efficacy may be particularly valuable in assessing patients’ preparedness to reduce or discontinue opioid use and identifying barriers and facilitators to change. In limited extant literature, factors such as lower pain severity and higher concern about opioids have been shown to predict greater readiness to taper (Crouch et al., 2023).

Acceptance and Commitment Therapy (ACT), a third wave behavioral therapy, appears to be a useful therapeutic approach for individuals with CP and comorbid substance dependence (Oveisi et al., 2024). ACT is conceptualized using the Hexaflex model, which highlights six key dimensions of value-based living through cognitive and behavioral approaches (Rolffs et al., 2018). One of these six dimensions is acceptance, referring to willingness to face unpleasant realities, which is contrasted with experiential avoidance, or diversion from things that are unpleasant or painful, physically or emotionally (Rolffs et al., 2018). The concept of acceptance has been applied to CP, with pain acceptance defined as openness to engaging with and tolerating pain without compromising valued elements of one’s life (LaChapelle et al., 2008).

The construct of pain acceptance appears to be particularly relevant to LTOT and CP. Greater levels of pain acceptance is associated with less functional impairment, pain interference, pain-related disability, depression, pain medication use, and opioid use (Kratz et al., 2018; Lin et al., 2015; White et al., 2024; Ye et al., 2024). ACT interventions that emphasize pain acceptance are also associated with lowered likelihood of developing both CP and engaging in LTOT post-surgery (Anthony et al., 2020; Dindo et al., 2018). ACT has effectively reduced pain response among individuals with comorbid pain and opioid use disorder (Smallwood et al., 2016). Although the research in this area is more limited, pain acceptance may also be a facilitator of readiness to change when it comes to opioid use for CP. Engagement in ACT treatments is associated with movement along the change spectrum among daily smokers (Mak et al., 2020), including those with CP (Oveisi et al., 2024).

While readiness to change and pain acceptance have been investigated in the contexts of pain and substance use disorder, with promising findings, to our knowledge these constructs have not been studied concurrently in LTOT patients, who typically demonstrate low motivation for engaging in nonpharmacological pain treatments (Becker et al., 2017). Exploration of risk and protective factors for opioid misuse and opioid tapering, and particularly malleable factors like pain acceptance, can inform prevention and treatment outcomes. Thus, this study aimed to investigate the relationship between pain acceptance and opioid misuse behaviors and readiness to change opioid use among a community sample of non treatment-seeking individuals with CP using opioid medications. We hypothesized that both dimensions of pain acceptance would be significant, protective predictors of opioid misuse, readiness to change, and confidence to change, above and beyond pain severity.

## Method

### Participants and procedures

A community sample of adults with a variety of CP conditions using opioid pain medications were recruited through ResearchMatch.org. In addition to having CP, defined as pain on at least half of the days for at least 3 months, and currently taking a prescribed opioid medication, eligible participants were required to be at least 18 years of age, speak English, and live in the United States. After providing informed consent, participants completed an online survey in REDCap that required approximately 30 min to complete. Given the that the survey was completed without researcher supervision, validity checks were used to verify attention to the questions and flag records for removal. Out of a total of 286 individuals that were screened, 102 did not complete the survey and 24 records were removed following prespecified validity checks before analysis, resulting in a total sample of *N* = 157. Participants were compensated with a $15 electronic gift card. The study was approved by the university’s Institutional Review Board.

### Measures

Pain acceptance was measured using the Chronic Pain Acceptance Questionnaire – Revised (CPAQ-R), a validated self-report measure (McCracken et al., 2004). The CPAQ is a 20-item scale with Likert-style responses ranging from 0 (*never true*) to 6 (*always true*), with some items being reverse-scored. It contains two subscales, Activities Engagement and Pain Willingness. Total scores range between 0 (*very low function and satisfaction*) and 120 (*high function and satisfaction*). Internal consistency for the current sample was good (*ɑ* = 0.89).

The Prescription Opioid Misuse Index (POMI) was developed to assess misuse of prescription opioids (Knisely et al., 2008). POMI is an 8-item self-report measure with yes/no responses. Total scores were calculated by adding affirmative responses, with two or more indicating likelihood of an opioid misuse diagnosis (Knisely et al., 2008). A total score was calculated as a measure of opioid misuse severity, and a cut point of two was used to create a dichotomous variable. Internal consistency was good in the present sample (*ɑ* = 0.80). The POMI was selected because it is a brief, validated screening instrument designed to identify opioid misuse behaviors among individuals prescribed opioid medications. Given the exploratory nature of the study and the broader survey battery, the POMI provided a pragmatic measure of misuse while minimizing participant burden.

Pain was assessed using the Brief Pain Inventory (BPI) (Cleeland and Ryan, 1994). The BPI consists of 32 questions with a range of response choices. It contains two subscales, pain severity and pain interference. Pain intensity is marked from 0 (*no pain*) to 10 (*pain as bad as you can imagine*). Pain interference is ranked from 0 (*does not interfere*) to 10 (*completely interferes*). The BPI has excellent test–retest reliability and internal consistency for CP populations (Erdemoglu and Koc, 2013). Internal consistency for the current sample was excellent (*ɑ* = 0.92).

A Visual Analog Scale (VAS) (Halfaker et al., 2011) was used to measure confidence and readiness to taper opioid use. Items were adapted from the readiness ruler often used as a clinical tool in motivational interviewing interventions. Participants were asked, “What is your readiness to decrease [not necessarily stop] opioid medication(s)?” and “What is your confidence in decreasing [not necessarily stopping] opioid medication(s)?” Response items ranged from 0 (not at all confident/ready) to 10 (extremely confident/ready).

### Data analyses

All analyses were conducted using SPSS version 29 (IBM SPSS Statistics, 2021). Because these were secondary analyses, we conducted sensitivity analyses based on the available complete-case samples. At *α* = 0.05 and 80% power, the linear regression models were adequately powered to detect an overall effect of *f*^2^ = 0.07, equivalent to approximately *R*^2^ = 0.065. For the logistic regression, the available sample provided 80% power to detect odds ratios of 0.52 or smaller for activity engagement and 0.49 or smaller for pain willingness, equivalent in magnitude to odds ratios of 1.93 or larger and 2.03 or larger, respectively, accounting for the other CPAQ subscale and pain severity. Accordingly, the analyses may have been insufficiently powered to detect smaller associations and should be interpreted as exploratory. Data were examined for statistical assumptions before all analyses. All assumptions were met and there were no concerns for multicollinearity (VIFs < 2). Using the Little MCAR test, missing data patterns were assessed, which confirmed low missing data (<5%) and data missing completely at random. Therefore, estimation maximization was used to impute missing data for main variables of interest. Descriptive statistics were reviewed in count/percentages for categorical variables and mean/SDs for continuous variables. Multiple regression analyses were conducted to predict opioid misuse, readiness to taper opioids, and confidence to taper opioids, respectively. Three independent variables, pain severity and each of the pain acceptance subscales, were added into the three models simultaneously. Predictors were selected *a priori*.

## Results

The sample consisted of 157 participants. The majority of participants were female (79%) and Caucasian (83%). Nearly 30% of participants had a primary diagnosis of chronic back pain. Two thirds of the sample (66.2%) had been experiencing pain symptoms for at least 10 years. More than half of the sample had an anxiety (52.9%) or depression (58.0%) diagnosis. Inclusion of gender in the regression models did not meaningfully alter the magnitude or statistical significance of the observed associations. Full descriptives for the sample can be found in Table 1.

**Table 1:** Sample demographics and medical characteristics.

Variable	*n* (%)	M(SD)
Age		51.6 (13.0)
Gender		
Female	124 (79.0)	
Male	28 (17.8)	
Gender Minority	4 (2.5)	
Not Reported	1 (0.6)	
Race^1^		
American Indian or Alaska Native	7 (4.5)	
Asian or Asian American	4 (2.5)	
Black or African American	22 (14.0)	
Multiracial	7 (4.5)	
White or caucasian	130 (82.8)	
Other	8 (5.0)	
Number of pain diagnoses		6.3 (3.0)
Number of pain areas		6.3 (3.3)
Pain duration		
<1 year	4 (2.5)	
1–5 years	18 (11.5)	
5–10 years	31 (19.7)	
10+ years	104 (66.2)	
Primary pain diagnosis type		
Back pain	46 (29.2)	
Arthritis	26 (16.6)	
Fibromyalgia	16 (10.2)	
Neuropathic	11 (7.0)	
Musculoskeletal	9 (5.7)	
Post-injury	9 (5.7)	
CRPS	8 (5.1)	
Headache	5 (3.2)	
Other	27 (17.2)	
Pain severity average (0–10)		5.8 (1.6)
Pain interference average (0–10)		6.2 (2.0)
Pain acceptance		56.6 (18.5)
Pain willingness		23.4 (9.5)
Activity engagement		33.2 (12.0)
Mental health diagnosis		
None	52 (33.1)	
Depression	91 (58.0)	
Anxiety	83 (52.9)	
Other	36 (22.9)	
Prescription opioid misuse index (POMI)		
Below opioid misuse cutoff	72 (70.6)	
Above opioid misuse cufoff	30 (29.4)	
Readiness to taper (0–10)		3.5 (3.4)
Confidence to taper (0–10)		3.8 (3.5)

^1^Multiple boxes could be selected for racial or ethnic identity, which included American Indian or Alaska Native, Asian or Asian American, Black or African-American, Native Hawaiian or Pacific Islander, White or Caucasian, Multiracial, and Other.

### Opioid misuse

A logistic regression was used to examine the association of pain severity and acceptance with opioid misuse. The sample size for this analysis was *n* = 102. The overall model predicted opioid misuse, Wald’s *χ*^2^(3) = 20.80, *p* < 0.001 with a Nagelkerke *R*^2^ of 0.26. Within the block, activity engagement, OR = 1.08 (95% CI: 1.03–1.14), *p* = 0.003, and pain willingness, OR = 0.89 (95% CI: 0.83–0.96), *p* = 0.002, were significant predictors (see Figures 1, 2). Pain severity was not a significant predictor, OR = 1.11 (95% CI: 0.79, 1.57), *p* > 0.05.

**Figure 1:**
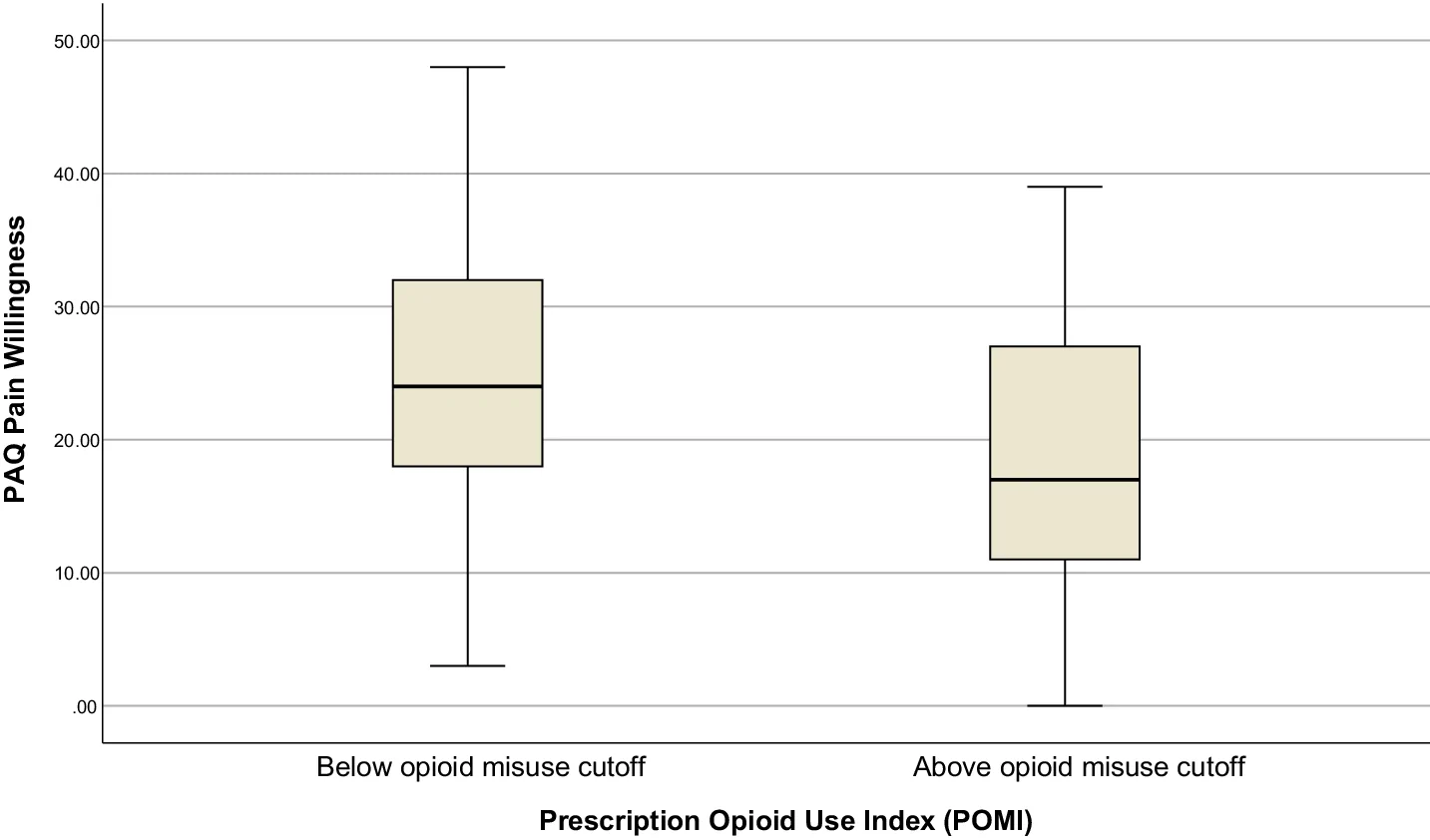
Pain willingness stratified by POMI classification.

**Figure 2:**
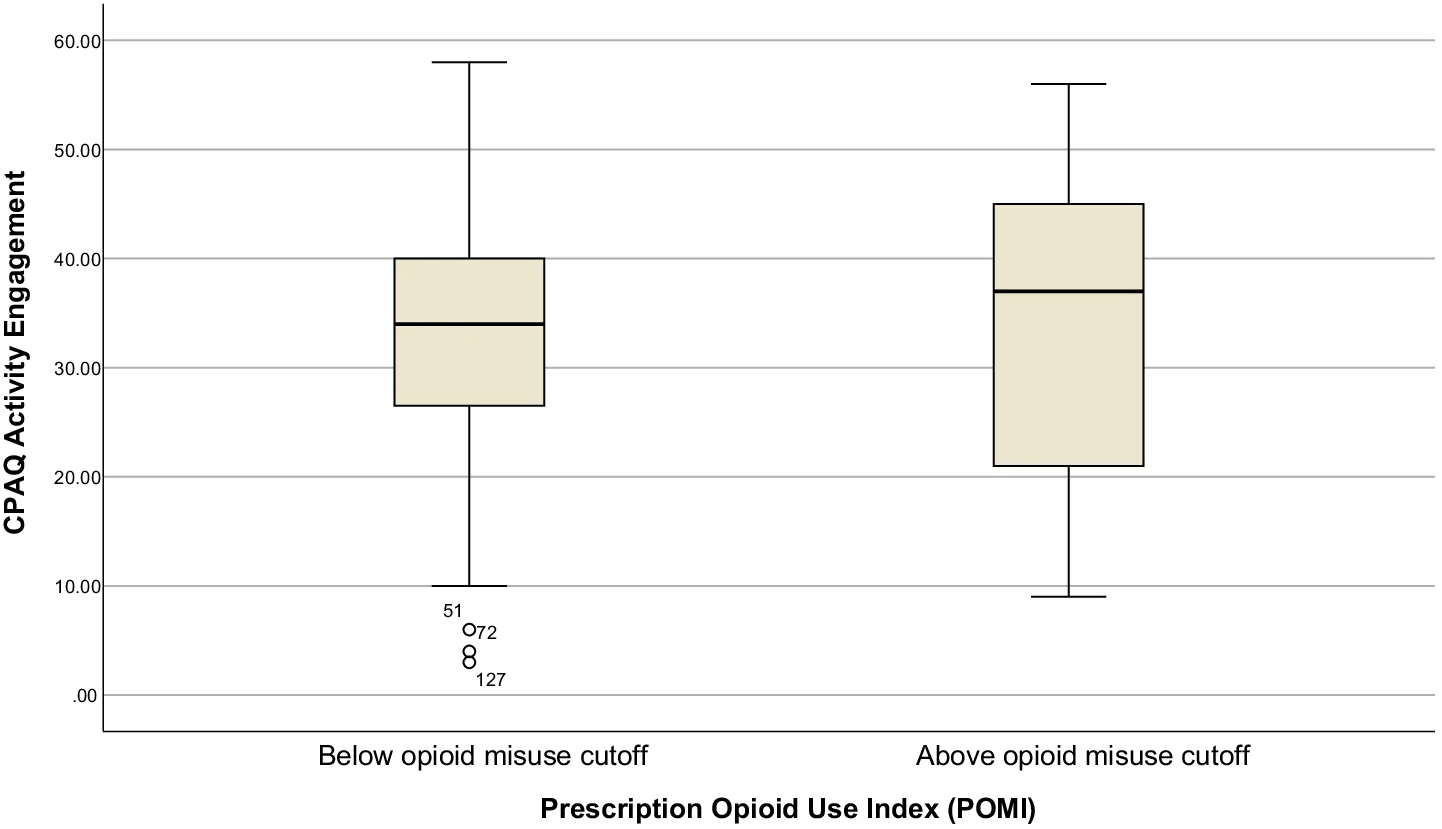
Activity engagement stratified by POMI classification.

### Readiness to reduce opioid use

A multivariate regression was used to examine the association of pain severity and acceptance with readiness to reduce opioid use. The sample size for this analysis was *n* = 156. The overall model predicted readiness to change, (*R*^2^ = 0.09, *F*(3, 153) = 4.88, *p* < 0.01). Activity engagement (*b =* 0.07, 𝛽 = 0.25, *p* < 0.01) was positively associated and pain willingness (*b* = −0.07, 𝛽 = −0.19 *p* = 0.04) was negatively associated with readiness to change. Pain severity was not a significant predictor (*b* = 0.32, 𝛽 = 0.16, *p* > 0.05).

### Confidence to reduce opioid use

A multivariate regression was used to examine the association of pain severity and acceptance with confidence to reduce opioid use. The sample size for this analysis was *n* = 154. The overall model predicted confidence to taper opioid use, (*R*^2^ = 0.06, *F*(3, 151) = 2.96, *p* < 0.01). Activity engagement (*b =* 0.07, 𝛽 = 0.25, *p* < 0.01) and pain willingness (*b* = −0.07, 𝛽 = −0.20, *p* = 0.04) were significant predictors. Pain severity was not a significant predictor (*b* = −0.09, 𝛽 = −0.04, *p* > 0.05).

## Discussion

This cross-sectional study investigated the relationship between CP acceptance and opioid-related outcomes among adults with CP using opioids, with the hypothesis that pain acceptance would be more predictive of outcomes than pain severity. As expected, pain acceptance was predictive of opioid misuse as well as readiness and confidence to taper opioid use, whereas the association between pain severity and opioid-related outcomes was not significant. However, disparate directionality in the relationship between each of the two subscales of pain acceptance and the outcomes was not hypothesized, adding nuance to our understanding of the role of pain acceptance among individuals on LTOT. Effect sizes were small, indicating that practical significance is somewhat limited, but significant findings suggest that further investigation is warranted.

Opioid misuse was negatively associated with pain willingness but positively associated with activity engagement in the present study, which is partially consistent with other studies suggesting that pain acceptance and related constructs are linked with lower levels of opioid misuse (Esteve et al., 2024; Rhodes et al., 2021; Smit et al., 2023). By breaking pain acceptance into its two factors for the purpose of our analyses, we were able to examine and identify differential effects. Pain willingness refers to the willingness to experience rather than avoid pain symptoms. As anticipated, greater equanimity regarding one’s pain was associated with less misuse of opioids (see Figure 1). Activity engagement, on the other hand, refers to the pursuit of meaningful life activities regardless of one’s pain levels. Unexpectedly, activity engagement was associated with greater likelihood of opioid misuse (see Figure 2). Because these data are cross-sectional, the direction of this association cannot be determined. One possibility is that opioid use, including patterns reflected in a positive POMI screen, enables some individuals to continue pursuing activities despite pain. Alternatively, the activity engagement subscale may not fully distinguish flexible, values-consistent engagement from more rigid patterns of persisting with activity despite worsening pain. Although overactivity has been associated with greater opioid use in prior research (Andrews et al., 2016), the CPAQ does not directly assess overactivity, and this interpretation therefore remains speculative. Longitudinal research incorporating specific measures of activity patterns and pacing is needed to clarify the mechanisms and directionality of this association.

Pain willingness and activity engagement were both associated with readiness and confidence to decrease opioid use. To our knowledge, this relationship between acceptance and readiness to change has not been studied in a CP population, but previous findings have suggested that pain acceptance is predictive of readiness to change among individuals with substance dependence (Oveisi et al., 2024). Value-based activity pursuit is associated with engagement in treatment (i.e., movement along stages of change) for both CP (Miró et al., 2018) and substance use (Berman and Kurlancheek, 2021). Since reductions in opioid doses might result in temporary increases in pain exposure, we might expect openness to facing pain to correspond with greater readiness and confidence to reduce opioid use. However, in the present sample, higher willingness to experience pain was associated with lower interest in and self-efficacy around change. Importantly, confidence and readiness were measured using a single-item scale, making it advisable to interpret this finding cautiously. If we are to postulate, it may be that pain willingness supports a realistic assessment of pain management needs and more measured approach, associated with appropriate use of opioids and thus a lower interest in tapering. An additional hypothesis is that higher activity engagement favors behavior change as the result of its association with greater opioid misuse. Another possibility is that someone who is pursuing valued activities in spite of pain may favor action in the face of barriers, thereby feeling prepared to taper opioids. It is perhaps not surprising that behavioral activation might lower reliance on substances for pleasure or relief, thereby promoting a belief that reducing opioid use is feasible.

Unlike pain acceptance, pain severity was not a significant predictor in any of our models, consistent with our hypothesis that pain acceptance would predict opioid misuse, readiness to change, and self-efficacy above and beyond pain severity. The literature shows associations between pain severity and higher-risk opioid use, greater opioid misuse, and higher withdrawal scores (Charron et al., 2022; Craft et al., 2025) meaning that pain severity may increase barriers to tapering; additionally, movement along the stages of change predicts lower pain severity (Mun et al., 2019). Consistent with these findings, pain severity in the present study was associated with opioid-related outcome measures without the introduction of pain acceptance measures into the model. Our results suggest that pain acceptance accounts for some variance previously explained by pain severity, making pain acceptance a more meaningful predictor.

In sum, both dimensions of pain acceptance, activity engagement and pain willingness, were significant predictors across all three models, but not exactly in the way that was expected. Pain willingness and activity engagement had opposite associations with opioid-related outcomes, which highlights the importance of assessing the factors individually. Furthermore, the results for opioid misuse did not correspond as expected with those for confidence and readiness to change, demonstrating flipped associations with pain acceptance factors, making it challenging to determine whether either factor is “protective.” Notably, readiness and confidence to change are metrics of intention, whereas opioid misuse is a behavior. It makes sense that one’s reported intentions may not be representative of de facto behavior; thus, it is possible for one to report a belief that one could reduce opioid use, but to not fully account for barriers or realistically assess the change process.

Overall, findings from this study suggest the relevance of pain acceptance when considering opioid risk behaviors and readiness to change among individuals with CP. The present study is the first to explore the association of pain acceptance and opioid use across a range of pain diagnoses and etiologies, and findings broadly align with previous disease-specific studies that found pain acceptance facilitates reduced opioid use following spinal cord injury (Kratz et al., 2018) and in chronic lower back pain (Smit et al., 2023). Not only has pain acceptance been shown to decrease pharmacological treatment for CP, it has also been found to directly reduce pain symptomology, including pain interference, pain intensity, and functional impairment (Ye et al., 2024). Because pain experience and opioid dependence are closely interrelated, with one often reinforcing the other (Ditre et al., 2019) it is possible that changes in pain perception also reduce medication reliance. Others have suggested that pain acceptance is effective in multiple domains because it improves emotion regulation, thus modulating the psychological and physiological pain responses and reducing the avoidance that may facilitate reliance on opioid medications (Aaron et al., 2020). More research is needed to understand the mechanisms of pain acceptance as it relates to opioid use and readiness to change in individuals with CP. There are a few notable limitations of the present study. Recruitment and data collection were all conducted virtually. Due to the absence of *a priori* power analyses for these analyses from the larger parent study, there is potentially limited power to detect smaller associations. Limited gender and racial representation in the sample reduce generalizability, making it difficult to extrapolate findings beyond Caucasian women. The cross-sectional design of the study limited assessment of directionality or causality. Finally, while the use of single-item measures for readiness and confidence to change lowered participant burden, they may have resulted in measurement error. Future studies should consider using a more robust, validated instrument to assess readiness and confidence to change.

## Conclusion

In a mixed CP sample of opioid users, the activity engagement factor of pain acceptance was a significant predictor of opioid misuse but also readiness and confidence to reduce opioid use, whereas pain willingness predicted less misuse but also less readiness and confidence to change. Findings suggest that pain acceptance is a nuanced construct that may play a unique role among individuals on LTOT with CP, which may influence the efficacy of ACT in this population. Future studies should compare components of pain acceptance among LTOT with CP and non-LTOT with CP and explore longitudinal associations between pain acceptance and stages of change in individuals receiving ACT interventions for CP.

## Data Availability

The raw data supporting the conclusions of this article will be made available by the authors, without undue reservation.
